# The Challenge of Lyme Borreliosis: Knowledge, Attitudes, and Practices in France

**DOI:** 10.3390/biology14091286

**Published:** 2025-09-17

**Authors:** Cynthia Philibert, Joana Ferrolho, Mark S. Gibson, Sérgio Ramalho de Sousa, Hugo Vilhena, Sofia Anastácio

**Affiliations:** 1 Department of Veterinary Sciences, Vasco da Gama University School, Avenida José R. Sousa Fernandes 197 Lordemão, 3020-210 Coimbra, Portugal; aluno.1700622@euvg.pt; 2 Vasco da Gama Research Centre (CIVG), Department of Veterinary Sciences, Vasco da Gama University School, Avenida José R. Sousa Fernandes 197 Lordemão, 3020-210 Coimbra, Portugal; joana.gibson@euvg.pt (J.F.); mark.gibson@euvg.pt (M.S.G.); sergio.sousa@euvg.pt (S.R.d.S.); hcrvilhena@hotmail.com (H.V.); 3 Department of Veterinary Clinics, School of Medicine and Biomedical Sciences, ICBAS-UP, University of Porto, 4050-313 Porto, Portugal; 4 Animal and Veterinary Research Centre (CECAV), University of Trás-os-Montes and Alto Douro (UTAD), Quinta de Prados, 5000-801 Vila Real, Portugal; 5 Associate Laboratory for Animal and Veterinary Science—AL4AnimalS, 1300-477 Lisboa, Portugal; 6 Center of Neurosciences and Cell Biology, Health Science Campus, 3000-548 Coimbra, Portugal

**Keywords:** Lyme borreliosis, *Borrelia burgdorferi*, dogs, veterinarians, knowledge–attitudes–practices, ticks, one health, France

## Abstract

Lyme borreliosis (LB), a zoonotic disease caused by *Borrelia burgdorferi* sensu lato and transmitted by *Ixodes ricinus*, is increasingly relevant in France, yet data on public awareness and prevention are limited. This cross-sectional study assessed the knowledge, attitudes, and practices (KAP) of companion-animal owners (CAOs) and veterinarians regarding LB. Online questionnaires were distributed between January and April 2023, yielding 159 valid CAO responses and 48 responses from veterinarians. Among owners, 95% reported outdoor access for their pets, 73.9% had observed ticks, and 88.8% used ectoparasiticides. However, only 16.4% reported LB vaccination, and just 38% recognized key canine clinical signs. Veterinarians frequently suspected LB (70%) but confirmed diagnoses less often (39.6%), relying mainly on clinical signs and the results of rapid tests. While prevention through antiparasitic use was common, communication between veterinarians and owners regarding LB was inconsistent. Despite this, most owners reported compliance with veterinary guidance. These findings reveal good preventive habits but identify knowledge gaps and underdiagnosis. Enhancing veterinary–client communication and promoting a One Health approach could improve LB prevention and control in France.

## Simple Summary

Lyme disease is an illness that spreads through tick bites and can affect both animals and people. In France, the disease is becoming more common, but the knowledge regarding people’s awareness—especially pet owners and veterinarians—is limited. In this study, we used online questionnaires to enquire with French dog and cat owners, as well as veterinarians, regarding their knowledge, and prevention and clinical management strategies of tick infestation and Lyme disease. Our results showed that most pet owners use tick prevention products and follow veterinarians’ advice. However, many do not recognize the signs of illness in dogs or have knowledge about vaccination. Veterinarians often suspect Lyme disease but laboratory confirmation is less frequent, usually relying on signs and the results of simple tests. One important problem identified in this research is the lack of communication about Lyme disease, which may may limit adequate prevention. Improvement of communication and education between veterinarians and pet owners could contribute to the better protection of pets and people. This study showed the importance of an interdisciplinary approach to animal, human, and environmental health—a concept known as “One Health”—to fight tick-borne diseases more effectively.

## 1. Introduction

Lyme disease (LD), or Lyme borreliosis, is a tick-borne zoonosis of growing concern in both human and veterinary medicine. First recognized in humans in the 1970s in the town of Lyme, Connecticut (USA), the disease was subsequently identified in dogs in 1984 and in cats in 1986 [1,2,3]. The causative agent, *Borrelia burgdorferi*, a spirochete bacterium discovered by Willy Burgdorfer in the 1980s, belongs to the order *Spirochaetales*, family *Borreliaceae*, and the *Borrelia burgdorferi* sensu lato (Bbsl) genospecies complex, of which only a few are known to be pathogenic. These include *B. burgdorferi* sensu stricto (*Bb*ss), prevalent in North America, and *B. afzelii* and *B. garinii*, which are predominant in Europe, including France [4,5].

The enzootic cycle of *B. burgdorferi* involves a broad range of vertebrate hosts, with small mammals and birds serving as primary reservoirs [6,7]. Transmission occurs via hard ticks of the genus *Ixodes*, particularly *Ixodes ricinus*, which is the principal vector in France. This species follows a three-host, two-year life cycle, with larvae feeding on small vertebrates, nymphs on medium-sized mammals, and adults on larger hosts [7,8]. For *Bb*sl transmission to occur, an infected tick must remain attached to the host for at least 24–52 h, with peak transmission at around 48 h. While transstadial transmission is the primary route, co-feeding may also contribute to pathogen spread; transovarial transmission, however, is considered negligible [3,4].

In Europe, the spatial distribution of *Bb*sl is closely aligned with that of *I. ricinus* [9], highlighting the importance of vector ecology in disease dynamics. In humans, LD manifests in three clinical stages: an early localized phase marked by the pathognomonic erythema migrans (a skin rash associated with LD observed in 60–80% of symptomatic patients), an early disseminated phase potentially involving neuroborreliosis or Lyme arthritis, and a late disseminated phase characterized by chronic neurological, articular, or dermatological involvement [6,8].

Estimating LD incidence across Europe remains challenging due to variability in surveillance systems and case definitions. Nonetheless, the incidence in Western Europe is estimated to range between 10 and 156 cases per 100,000 inhabitants annually [10]. In France, the disease is reported nationwide, with an increasing trend in confirmed cases—from 46 per 100,000 in 2009 to 84 per 100,000 in 2016 [11]. Humans are considered incidental hosts, with infections frequently being linked to outdoor activities in endemic areas [6]. However, only 10–40% of individuals bitten by an infected tick develop clinical illness [4].

In companion animals, diagnosing LD is hindered by the high prevalence of subclinical infections—only around 5% of seropositive dogs develop clinical signs [1]. Moreover, the absence of systematic surveillance in pets limits accurate estimations of prevalence. In North America, seroprevalence studies in 2022 estimated rates of 3.8% in dogs in the United States and 3.3% in Canada [12]. In France, published studies report seroprevalence ranging from 1.1% to 25%, with an average estimate of 12.2% [13,14].

Although dogs do not directly transmit LD to humans, pet ownership has been shown to increase human exposure to ticks and associated pathogens [15,16]. Consequently, dogs have been proposed as effective sentinels for human risk. Serological studies in dogs are practical and informative, with data correlating canine seroprevalence to human incidence—especially in transitional or emerging areas of endemicity [17,18]. Given the shared environmental exposure and similar risk factors, the use of dogs as sentinels offers a valuable tool for monitoring the geographic spread of LD and anticipating human outbreaks.

The eco-epidemiological complexity of LD necessitates a One Health approach to disease control, integrating human, animal, and environmental health. Prevention in companion animals relies primarily on vector control, especially through the regular use of external antiparasitic agents. Although LD vaccines are available for dogs in France, their use remains limited and somewhat controversial, owing to questions regarding efficacy, target populations, and owner awareness [3,19].

Understanding the knowledge, attitudes, and practices (KAP) of both pet owners and veterinarians is essential for guiding effective prevention and control strategies. While a Canadian study indicated that most veterinarians feel confident managing LD, it also revealed variability in knowledge depending on clinical experience [20].

This study aimed to evaluate the knowledge, attitudes, and practices of companion-animal owners and veterinarians regarding LD in the French context, where LD is endemic, but companion-animal data remain scarce. The goal was to identify strengths and gaps that can inform targeted educational strategies and reinforce the One Health framework in LD management.

## 2. Materials and Methods

### 2.1. Study Design

To achieve the research objectives, a descriptive cross-sectional observational study was conducted in mainland France, a region where LD is endemic. For this purpose, two anonymous self-administered questionnaires, never published or used before, were prepared and distributed online (i) to companion-animal owners (CAOs)—focusing on dog and cat owners—residing in mainland France and (ii) to companion-animal veterinarians (VETs) actively involved in clinical practice across mainland France.

### 2.2. Ethics Committee Approval

The study protocol was approved by the Ethics Committee of Escola Universitária Vasco da Gama, Coimbra, Portugal (Approval No. 24/2022).

### 2.3. Questionnaires

The two questionnaires were crafted (November and December 2022) and initially pre-tested with ten CAO volunteers (dog and cat owners) and four VETs. These questionnaires underwent further refinement for clarity and relevance as needed. The language and format of the questions were kept simple and objective. Furthermore, the questions were designed with closed answers (i.e., yes/no) or as multiple choice. The VET questionnaires also included some open-ended questions to ensure more accurate data (e.g., animal species diagnosed with LD, clinical signs that raise the suspicion of LD).

Both questionnaires started with an introduction, where participants were informed of the purpose and the design of the study and informed that participation was voluntary. Also, it included a statement informing the participants of the anonymity of the data, their exclusive use for study purposes, and the estimated time to complete the questionnaire.

The validated questionnaires (Questionnaire S1 and Questionnaire S2) were inserted into the Google Forms^®^ platform (Google, Mountain View, CA, USA) and subsequently disseminated via multiple social media platforms [e.g., Facebook^®^ (Meta, Menlo Park, CA, USA), Instagram^®^ (Meta, Menlo Park, CA, USA), LinkedIn^®^ Microsoft, Redmond, WA), and X^®^ (X Corp, Bastrop, TX, USA). To increase the number of respondents, QR codes were generated (https://www.qr-code-generator.com/, accessed 22 January 2023), embedded in two posters (one for CAOs and the other for VETs) and displayed in veterinary practices to encourage participation. The VET questionnaire was also shared through Vetofocus^®^, a collaborative website for veterinarians and veterinary students in France. An additional dissemination strategy was implemented for the VET group, involving direct email distribution to veterinary clinics and hospitals. Due to the voluntary participation based on online dissemination, representativeness may have been affected by biases from self-selection and access to digital media.

Data collection occurred from 30th January to 16th April 2023 for CAOs, and until 23rd April 2023 for VETs.

#### 2.3.1. Companion-Animal Owners (CAOs)

The CAO questionnaire, consisting of 34 questions, was organized into four sections after the introduction. Section I, “validation”, asked for confirmation of acceptance to participate in the study and informed consent regarding the use of the data obtained. Section II, “companion-animal owner”, aimed to characterize the CAO study group. Section III, “animal”, aimed to understand the habits of CAOs in relation to their pets. This section was focused on dogs and cats, which were considered to be the most common pets. Section IV, “Lyme Disease”, addressed CAOs’ perceptions about LD. CAO respondents without cats or dogs were redirected from section II to section IV.

Therefore, the structure was adaptive based on the species owned, and variables were included to characterize animal profiles, lifestyles, and owner preventive practices.

#### 2.3.2. Companion-Animal Veterinarians (VETs)

The VET questionnaire, comprising 29 questions, also consisted of four sections after the introduction. Section I, “validation”, asked for confirmation of acceptance to participate in the study and informed consent regarding the use of the data obtained. Section II, “veterinarians”, aimed to characterize the study group and to analyze their perception and habits in relation to ticks. Section III, “Lyme disease—dogs and cats”, addressed the knowledge and practices regarding LD, and finally, section IV addressed the communication strategy with the target audience.

### 2.4. Study Variables

To accomplish the objectives outlined for this study, variables were analyzed to characterize the two study groups and to obtain the information required for the study (Supplementary File S1).

#### 2.4.1. Independent/Descriptive Variables

Descriptive variables were aimed at characterizing of the study group. For CAOs, these were gender, age, education level, socioprofessional group, region, area of residence (urban/rural), housing type, and animal ownership (Table S1 Supplementary File S1).

Regarding VETs, these were gender, age, years in practice, the geographic region(s), and area(s) of clinical activity (Table S2 Supplementary File S1).

#### 2.4.2. Outcome Variables

Outcome variables were grouped according to the study’s key objectives: (i) characterization of habits among CAOs and VETs; (ii) evaluation of knowledge and practices regarding Lyme disease; (iii) assessment of communication strategies between VETs and CAOs; and (iv) evaluation of general Lyme disease knowledge among CAOs. A list of variables, classification, attributes and codes was prepared (from Tables S3–S13, Supplementary File S1).

### 2.5. Data Analysis

In this study, descriptive and statistical analysis were performed. Responses were analyzed quantitatively and qualitatively, reported as absolute frequency (n) and percentage (%). Responses to open-ended questions were analyzed and categorized. Data were subjected to descriptive statistical analysis using EpiInfo software (CDC, version 7.2.5.0). Additional statistical testing was performed using VassarStats (web version 2022), including the Chi-square test and Fisher’s exact test to assess significance. Results were considered statistically significant at *p* < 0.05 and an odds ratio (OR) > 1.

## 3. Results

### 3.1. Companion-Animal Owners

During the period of data collection, a total of 160 responses were obtained from companion-animal owners (CAOs) but one response was excluded due to missing data, resulting in 159 valid entries.

**Figure 1:**
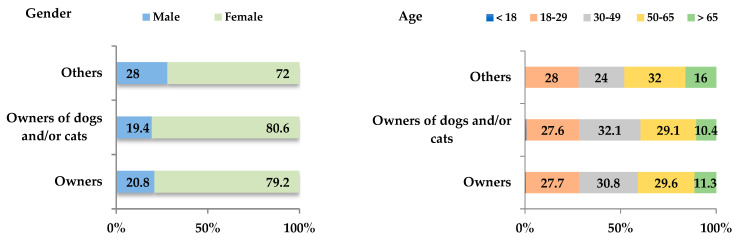

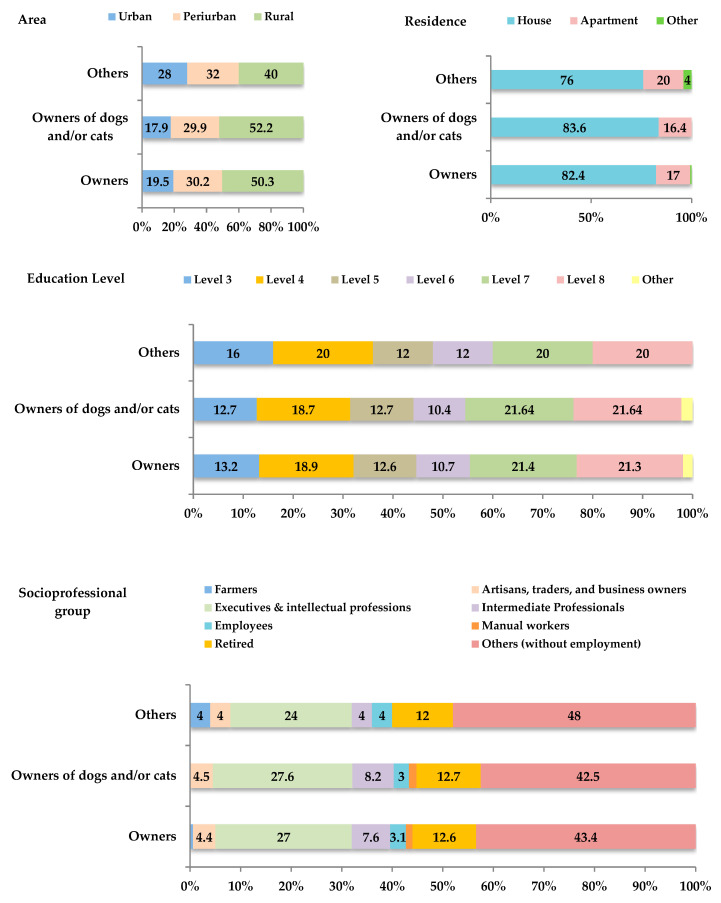
Characterization of respondents to the “companion−animal owners” questionnaire (n = 159).

The demographic characteristics of respondents are presented in Figure 1. Among CAO participants, 79.2% (n = 126; 95% CI: 72.1–85.3%) were female, 84.3% (n = 134; 95% CI: 77.7–89.6%) reported owning one or more dog and/or cat, 50.3% lived in rural areas (n = 80; 95% CI: 42.3–58.3%), 82.4% lived in a house (n = 131; 95% CI: 75.4–87.8%), and 70.4% (n = 112; 95% CI: 62.7–77.4%) resided in the Auvergne–Rhône-Alpes region. Thus not all metropolitan regions of France were equally represented among CAO respondents.

Regarding pet ownership, 84.3% (n = 134; 95% CI: 77.5–89.4%) of CAOs mentioned owning dogs and/or cats as pets; specifically, 31.5% (n = 50; 95% CI: 24.3–39.3%) reported having only cats, 30.2% (n = 48; 95% CI: 23.2–38.0%) reported having only dogs, and 22.6% mentioned having both (n = 36; 95% CI: 16.4–29.9%), while the remaining CAOs reported having other pets besides dogs and/or cats.

**Figure 2:**
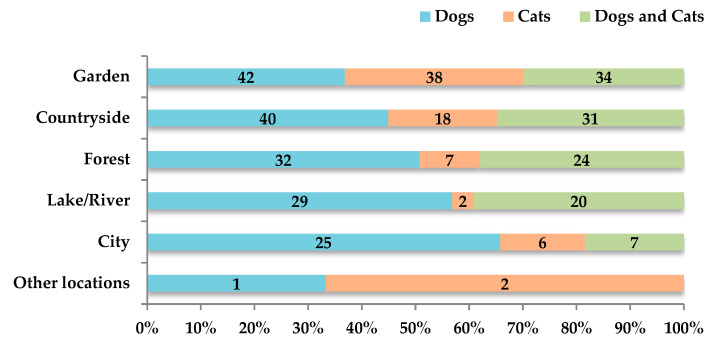
Distribution of outdoor environments visited by companion animals (dogs, cats, or both) with outdoor access in mainland France (n = 127). Data represent the number of reports from dog and/or cat owners regarding the types of environments their pets frequently accessed. Categories include garden, city, forest, lake/river, countryside, and other locations.

To analyze the pet care practices and habits of dog and/or cat owners, only this group of CAOs (n = 134) was considered. We observed that 94.8% (n = 127; 95% CI: 89.5–97.9%) of respondents reported that their animals had outdoor access. Outdoor access was reported in a balanced distribution by the owners of dogs (n = 48; 38%; 95% CI: 29.4–46.8%), cats (n = 43, 34%; 95% CI: 25.7–42.8%), and dogs and cats (n = 36, 28%; 95% CI: 20.7–37.0%). According to their owners, the place most frequented by dogs and/or cats is the garden (n = 114; 89.8%; 95% CI: 82.8–94.2%), followed by the countryside (n = 89; 70.1%; 95% CI: 61.2–77.7%) (Figure 2). Furthermore, according to their owners, 59.8% (n = 76; 95% CI: 50.8–68.4%) of these animals with outdoor access engaged in shared outdoor activities with their owners, such as hunting or walking in the forest.

**Figure 3:**
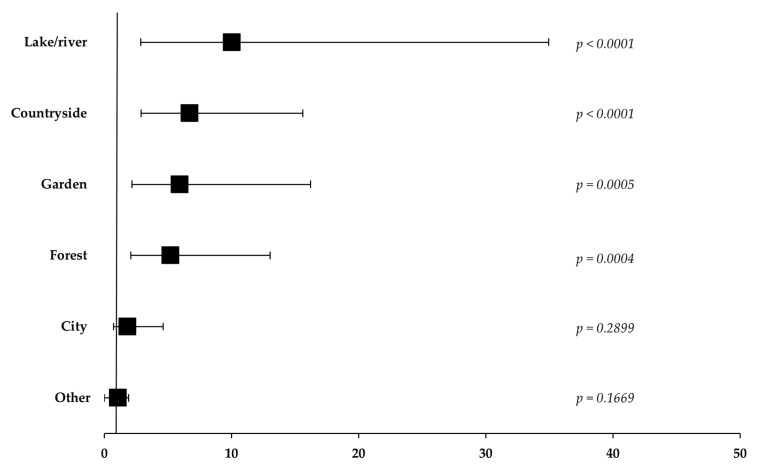
Analysis of the association between different factors and the observation of ticks in dogs and cats (n = 134). Time spent outdoors is associated with the presence of ticks on dogs and cats. Forest plots depicting the odds ratios (ORs) calculated for specific outdoor locations visited are shown as black squares. Horizontal bars show the 95% confidence interval (CI), with the respective *p*-values for each of the categories displayed adjacently.

The presence of ticks on dogs and/or cats was reported by 73.9% of owners (n = 99; 95% CI: 65.7–81.1%). All of the owners with dogs and/or cats without outdoor access reported not having encountered ticks on their animals. Thus, it was found that the likelihood of finding ticks on dogs and/or cats seems to be higher if they have outdoor access, particularly those spending time in rural areas (*p* < 0.0005) (Figure 3). Furthermore, and despite the limitations related to the study design and sample size, the engagement of dogs and/or cats and their owners in outdoor activities seems to act as an indicator of observing ticks on these pets (OR = 11.67; *p* < 0.0001).

The observation of ticks on pets was reported by 73.9% (n = 99; 95% CI: 65.5–80.9%) of owners. Additionally, approximately two-thirds of owners (66.4%; n = 89; 95% CI: 57.8–74.3%) believed that they lived in a tick-abundant area. Tick removal was reported by 69.4% of respondents (n = 93; 95% CI: 60.9–77.1%) whose animals all had outdoor access. Although careful interpretation of the results is required, tick removal was associated with outdoor activity (OR = 11.2; *p* < 0.0001). The preferred method of removing ticks was the use of dedicated tools (83.9%; 95% CI: 74.8–90.7%), while the remainder used fingers—with or without chemical substances such as ether or alcohol (16.1%; 95% CI: 9.3–25.2%).

**Figure 4:**
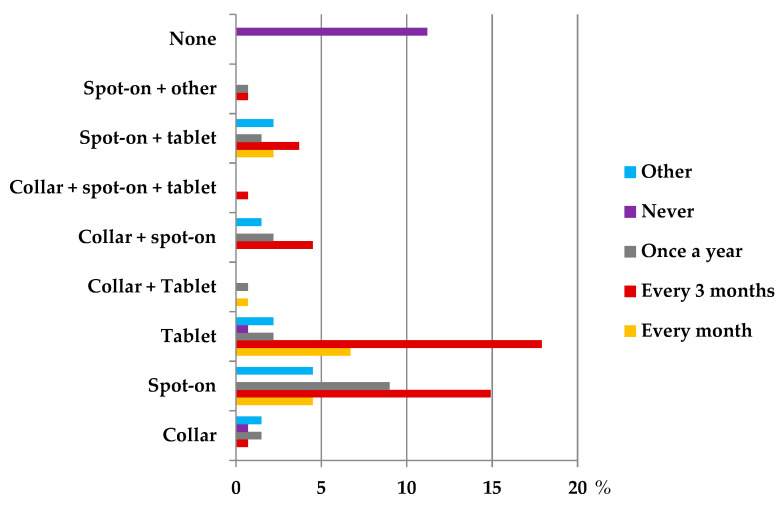
Type and frequency of external antiparasitic products (EAPs) used in companion animals, according to owners, as a percentage.

Regarding preventive measures, spot-on pipettes were the external antiparasitic products (EAPs) most mentioned by owners (32.8%; 95% CI: 25.0–41.5%), followed by oral tablets (29.9%; 95% CI: 22.3–38.4%). Additionally, the most common administration frequency indicated by respondents was quarterly (43.3%; 95% CI: 34.8–52.1%) (Figure 4). In this study, the use of EAPs showed a significant association with outdoor access (OR = 7.19; *p* < 0.05) and previous tick observation (OR = 5.37; *p* < 0.005).

It was noted that 81.3% (n = 109; 95% CI: 73.7–87.6%) of the owners reported following their veterinarian’s vaccination recommendations. Vaccination against LD was reported by 16.4% of respondents (n = 22; 95% CI: 10.6–23.8%), predominantly among owners who indicated they were following veterinary guidance (19.3%, n = 21; 95% CI: 12.3–27.9%).

Knowledge about LD was assessed among all CAOs (n = 159), revealing that 92.5% of respondents believe that they know how LD is transmitted (n = 147; 95% CI: 86.9–95.9%), and 91.8% (n = 146; 95% CI: 86.1–95.4%) correctly identified the transmission route, identifying ticks as the vector for the causative agent. Furthermore, 78.6% (n = 125; 95% CI: 71.3–84.6%) of respondents recognized that the causative agent of LD is not transmitted by all ticks.

When questioned about the clinical signs of LD in dogs, 42.1% (n = 67; 95% CI: 34.4–50.2%) of CAOs said they did not know what these were. Despite this, 23.9% (n = 39; 95% CI: 17.7–31.4%) identified joint symptoms, 20.1% (n = 32; 95% CI: 14.4–27.4%) identified neurological signs, 5% (n = 8; 95% CI: 2.4–10.0%) identified urinary signs, and 1.3% (n = 2; 95% CI: 0.2–4.9%) identified cardiac signs. Other clinical signs such as fatigue, loss of appetite, pale mucous membranes, and skin rash were specified by 3.1% (n = 5; 95% CI: 1.2–7.6%) of respondents. Moreover, an asymptomatic pattern was recognized by 4.4% (n = 7; 95% CI: 1.9–9.2%) of CAOs.

Regarding LD prevention measures, 37.7% (n = 60; 95% CI: 30.3–45.8%) of CAOs cited the combination of EAPs and physical inspection as the most effective strategy, which were followed by the combination of EAPs and vaccination (20.1%, n = 32; 95% CI: 14.4–27.4%), EAPs (13.2%, n = 21; 95% CI: 8.6–19.7%), vaccination (11.3%, n = 18; 95% CI: 7.0–17.6%), and physical inspection (10.6%, n = 16; 95% CI: 6.0–16.1%).

Regarding communication, 64.2% (n = 102; 95% CI: 56.2–71.6%) of CAOs reported never having discussed LD with their veterinarian. Of those who had (n = 57), 87.7% (n = 50; 95% CI: 76.3–94.9%) indicated that the conversation occurred during routine consultations. Among all of the CAOs, five reported that a case of LD has been diagnosed previously in one of their pets.

Overall, 96.2% (n = 153; 95% CI: 92.0–98.6%) of respondents acknowledged the link between ticks and LD. An awareness of LD in animals and humans was reported by 78.6% (n = 125; 95% CI: 71.4–84.7%) and 95.6% (n = 152; 95% CI: 91.1–98.2%) of respondents, respectively.

### 3.2. Companion-Animal Veterinarians

**Figure 5:**
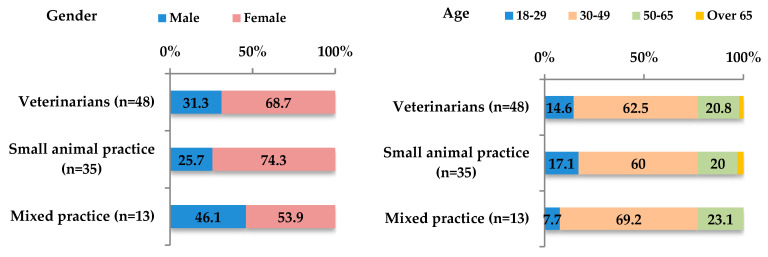

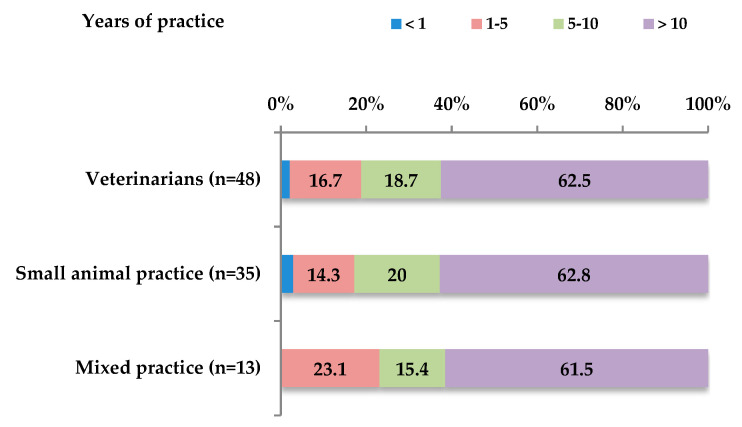
Characterization of respondents to the companion−animal-veterinarian questionnaire (n = 48).

Of the 50 companion-animal-veterinarian responses, two were excluded (one due to incomplete data and one due to ineligibility), resulting in 48 valid responses. The majority practiced canine medicine (72.9%, n = 35; 95% CI: 58.2–84.7%) and had over 10 years of clinical experience (62.5%, n = 30; 95% CI: 47.4–76.1%) (Figure 5). All regions of mainland France were represented, except Centre-Val de Loire, and the Auvergne–Rhône-Alpes region had the highest representation (25%, n = 12; 95% CI: 13.6–39.6%). It was observed that 85.4% (n = 41; 95% CI: 77.2–93.9%) of respondent veterinarians considered their practice area to be tick abundant. Interestingly, 75% (n = 36; 95% CI: 60.1–85.6%) reported never identifying ticks during clinical visualization. No relation was found between this finding and years of experience or practice area.

A suspicion of LD in dogs or cats during 2021–2022 was reported by 68.8% of veterinarians (n = 33; 95% CI: 53.6–80.9%), though no significant association was found with professional experience. Of these, 29 had performed diagnostic testing, most of whom (62.1%; n = 18; 95% CI: 42.4–78.7%) conducted between one and five tests during 2021–2022. The performance of diagnostic testing was significantly associated with clinical suspicion (OR = 5.33; *p* < 0.05). This analysis needs to be carefully considered due to limitations on sampling size.

The diagnosis of at least one case of LD in 2021–2022 was reported by 39.6% of veterinarians (n = 19; 95% CI: 26.1–54.7%), and an association between suspicion and diagnosis was observed (OR = 16.8; *p* < 0.005). Despite the low number of responses (n = 17 valid responses) regarding species in which LD has been diagnosed, it occurred mainly in dogs (76.5%, n = 13). One veterinarian reported the diagnosis in both dogs and cats.

Clinical suspicion was most commonly based on patient history and compatible signs (60.4%, n = 29; 95% CI: 45.3–74.2%). When asked about the clinical signs suggestive of LD, the most frequently cited clinical signs included arthritis (64.6%) and fever (62.5%). Other signs reported included lethargy; lymphadenopathy; tremors; hematologic abnormalities; anorexia; erythema; renal or cardiac signs; myopathy; and, in a few cases, uveitis or asymptomatic seropositivity.

Screening for tick-borne diseases was not conducted as a routine practice. In fact, 60.4% (n = 29; 95% CI: 45.3–74.2%) of veterinarians mentioned that they did not perform routine screening for tick-borne diseases, and 66.7% (n = 32; 95% CI: 51.6–79.6%) stated that did not propose testing for LD unless clinically indicated. Among the diagnostic tools used, the rapid qualitative serological test (e.g., SNAP 4Dx Plus Test^®^, Iddex, Westbrook, ME, USA) was the most prevalent (39.6%), even by some respondents who did not routinely test for LD.

LD was considered to be a differential diagnosis for recurrent or intermittent arthritis by 91.5% (n = 44; 95% CI: 80.0–97.7%) of veterinarians. Antibiotic therapy was primarily recommended when clinical signs coincided with seropositivity (70.8%; n = 34; 95% CI: 55.9–83.1%). Only 6.3% (n = 3; 95% CI: 1.3–17.2%) considered tick presence alone as being sufficient to initiate treatment. One respondent reported never prescribing antibiotics due to the lack of clinical suspicion, while another prescribed treatment in all suspected cases.

Regarding prevention, the use of EAPs alone or in combination with vaccination was reported by 35.4% (n = 17; 95% CI: 22.2–50.5%) of veterinarians, while 29.2% favored combining EAPs with animal inspection.

Concerning vaccination, 66.7% (n = 32; 95% CI: 51.6–79.6%) of veterinarians supported its use, and 60.4% (n = 29; 95% CI: 45.3–74.2%) highlighted its importance in high-risk animals. Among those who opposed vaccination (n = 16), the cited reasons were the asymptomatic nature of LD in dogs (n = 4), a perceived low disease prevalence (n = 4), the preference for EAPs (n = 3) as a preventive measure, a perceived low efficacy of the vaccine (n = 3), and a lack of information about the vaccine (n = 1). One respondent did not mention any reason.

Communication practices were evaluated through three targeted questions. Two-thirds of veterinarians (66.7%) reported discussing LD with clients—mainly during routine visits (34.4%), when ticks were detected (28.1%), or upon clinical suspicion of LD (34.4%). One respondent indicated engaging in all three scenarios. Despite these efforts, veterinarians rated their clients’ level of awareness as low, with a mean score of 1.77 on a 1–5 scale.

## 4. Discussion

This study presents valuable insights into the current knowledge, attitudes, and practices of companion-animal owners and veterinarians regarding Lyme disease (LD) in mainland France. Nonetheless, several limitations must be acknowledged. The exclusive use of online questionnaires and their strong dissemination by social media and e-mail may have introduced selection bias, favoring individuals with internet access and digital literacy. Moreover, the display of posters with QR codes in veterinary clinics may not have been uniform across the country which can explain the unbalanced geographic distribution of responses, limiting regional comparability. Furthermore, the limited sample size in each study group together with the convenience sampling, may compromise the accuracy and representativeness of the results obtained in this study.

In France, the primary vector of *Bb*sl, *I. ricinus*, is widespread, although its distribution and abundance are strongly influenced by environmental variables [7]. A national habitat suitability map published in 2022 provides an overview of regions at higher ecological risk [21]. In this study, a high proportion of respondents (73.5%) were from regions with high human LD incidence—Auvergne–Rhône-Alpes, Grand Est, and Nouvelle-Aquitaine—where the incidence exceeds 100 cases per 100,000 inhabitants according to the Sentinelles Network [22,23]. However, the low number of responses from other regions precluded an evaluation of regional differences in the perception of tick abundance.

Owners most frequently observed ticks in peri-urban forests and gardens, which corroborates previous findings [4]. Pet ownership—particularly of animals with outdoor access—increases the risk of exposure to ticks and tick-borne pathogens [7,16], a trend further exacerbated by shared outdoor activities [4].

Although 84% of respondents reported using appropriate tick removal tools (tweezers), a notable proportion (16%) employed non-recommended methods. The use of fingers or chemical substances may provoke salivary regurgitation, increasing *Bb*sl transmission risk [7]. In line with ACVIM recommendations, early removal using dedicated tick tweezers remains as the gold standard [19].

Vaccination against LD is not part of the core immunization protocols for dogs [24], which explains the low frequency of reported vaccination. Currently, only one LD vaccine is licensed for dogs in France (Merylim 3^®^ Boehringer Ingelheim, Ingelheim, Germany) [25]. Reports of feline vaccination likely reflect misperception or confusion among owners.

Most respondents (79.9%) believed a single tick bite was sufficient for transmission, despite the fact that the majority of infections remain subclinical—up to 95% in dogs and 60–90% in humans [1,6]. The perception of risk may also be influenced by the regional variability in tick infection rates [7], a notion echoed in our results (78.6%).

Although respondents correctly identified joint and neurological symptoms as indicative of LD in dogs, the most frequently reported clinical manifestations are articular and renal [19]. These discrepancies may suggest confusion between canine and human presentations. Nevertheless, most owners reported adherence to ACVIM-recommended preventive measures, namely the use of ectoparasiticides and regular inspection, reflected in the high proportion (88.8%) of respondents using external antiparasitic products (Figure 4).

One of the most notable findings was a perceptible gap in communication between veterinarians and pet owners regarding LD. The One Health framework underscores the pivotal role of veterinarians in zoonosis prevention and education [26,27], particularly in promoting tick control and disease awareness [7].

Despite this, a discrepancy emerged: while veterinarians considered owners to be poorly informed, many owners reported feeling knowledgeable about LD. Curiously, some respondents who initially claimed no awareness of LD transmission, later reported prior knowledge, suggesting possible recall or response bias.

In the veterinarian sample, regional representation was again uneven. Nonetheless, in the well-represented Auvergne–Rhône-Alpes region—all veterinarians acknowledged a high tick abundance, consistent with environmental suitability models [21].

In contrast to Canadian findings [20], most French veterinarians did not identify ticks during consultations. This finding requires further investigation among French veterinarians to understand whether this is due to limited training for tick identification, time constraints during clinical practice, or a lack of awareness of its diagnostic value. In fact, the distribution of ticks changes over time, challenging veterinarians to create evidence-based recommendations for tick prevention and to determinate appropriate testing and treatment protocols regarding tick-borne diseases [20].While most veterinarians reported suspecting LD, fewer had confirmed cases (39.6%). This may stem from diagnostic challenges, including the latency in seroconversion (4–6 weeks post-infection) and the non-specificity of clinical signs, which can overlap with other tick-borne illnesses [3,28]. Only one confirmed feline case was reported, which aligns with the literature highlighting the rarity of clinically overt LD in cats [19].

French veterinarians appeared to follow current diagnostic recommendations, basing suspicion on compatible clinical signs and documented tick exposure [28]. However, only two explicitly mentioned that most seropositive dogs are asymptomatic [3,28], perhaps reflecting limitations in the way questionnaire items were perceived.

Routine screening for LD was not standard practice. Two-thirds of veterinarians reported offering screening only when clinically indicated, despite ACVIM guidance recommending annual testing in asymptomatic dogs residing in endemic areas [19].

The rapid qualitative serological test (e.g., *SNAP* 4Dx Plus Test^®^, Iddex) emerged as the most commonly used diagnostic tool. Although some veterinarians mentioned the polymerase chain reaction (PCR), its use is discouraged due to its limited sensitivity in tissues, high cost, and technical demands [1,19]. Instead, serological testing—particularly ELISA-based methods such as the SNAP test—is preferred for assessing exposure, given its higher specificity compared to immunofluorescence (IF) assays [1,19].

Therapeutic decisions should be based on the presence of both clinical signs and seropositivity [3]. In accordance with best practices, doxycycline remains the first-line treatment, typically administered over four weeks [1,19].

Preventive strategies are centered on vector control. Current evidence supports the combined use of ectoparasiticides and physical inspection as the most effective approach [3,19]. Although many veterinarians recognized this, a subset favored ectoparasiticides alone or in combination with vaccination. This preference may reflect concerns about vaccine efficacy and the high prevalence of asymptomatic infections. Vaccination is currently recommended only for dogs at elevated risk in endemic regions and should be avoided in cases of suspected or confirmed infection [19].

Finally, communication remains a cornerstone of LD prevention. While most veterinarians believed that they communicated adequately with clients, they simultaneously rated owner awareness as low. Owners, conversely, often felt underinformed by their veterinarians despite possessing general knowledge about LD. This divergence may be explained by sample heterogeneity and differences in how information is delivered and perceived.

## 5. Conclusions

This study underscores the complex interplay between awareness, preventive behaviors, and professional guidance in the context of Lyme borreliosis in companion animals in mainland France. While most pet owners demonstrated responsible practices in tick prevention and removal, notable knowledge gaps persist, particularly regarding the clinical manifestations of the disease in dogs. Conversely, veterinarians were generally aligned with the current recommendations but reported limited diagnostic confirmation, suggesting possible under recognition or diagnostic challenges in clinical practice. A recurrent theme across both groups was the discordance between perceived and actual communication: veterinarians believed owners were poorly informed, yet most owners reported adherence to veterinary advice. This highlights a critical opportunity to strengthen veterinary–client dialogue.

Ultimately, veterinarians occupy a pivotal role at the human–animal–environment interface and must be empowered to lead educational efforts within a One Health framework. Bridging these gaps through improved communication and targeted outreach will be essential to mitigate the impact of Lyme borreliosis and enhance disease surveillance, prevention, and control in both animals and humans.

## Data Availability

The original contributions presented in this study are included in the article/Supplementary Materials. Further inquiries can be directed to the corresponding author.

## Supplementary Materials

The following supporting information can be downloaded at: https://www.mdpi.com/article/10.3390/biology14091286/s1, Table S1: Characterization of Veterinarians Regarding Practices Related to Lyme Disease (LD); Table S2. Descriptive variables for veterinarian group characterization; Table S3. Characterization of Companion Animal Owners Based on Pet Outdoor Access and Activity Patterns; Table S4. Characterization of Individuals Based on Tick-Related Practices; Table S5. Determination of Preventive Measures Used by the Study Group; Table S6. Characterization of Veterinarians Regarding Tick-Related Habits; Table S7. Assessment of Public Knowledge Regarding the Epidemiological Role of Ticks in Lyme Disease (LD); Table S8. Assessment of Public Knowledge Regarding Lyme Disease (LD) in Companion Animals; Table S9. Preventive Measures and Attitudes Toward Vaccination in the Study Group; Table S10. Characterization of Veterinarians Regarding Practices Related to Lyme Disease (LD); Table S11. Evaluation of Communication and Exposure to Information About Lyme Borreliosis, by CAOs; Table S12. Evaluation of Veterinarian Communication Regarding Lyme Borreliosis; Table S13. Assessment of Public Knowledge Regarding Lyme Disease (LD) in general; Questionnaire S1; Questionnaire S2.

## Abbreviations

The following abbreviations are used in this manuscript:

ACVIM	American College of Veterinary Internal Medicine
ANSES	Agence nationale de sécurité sanitaire de l’alimentation, de l’environnement et du travail
ARA	Auvergne–Rhône-Alpes
*Bb*sl	*Borrelia burgdorferi* sensu lato
*Bb*ss	*Borrelia burgdorferi* sensu stricto
CAOs	Companion-animal owners
CI	Confidence interval
ELISA	Enzyme-linked immunosorbent assay
GES	Grand Est
*I. ricinus*	*Ixodes ricinus*
IF	Immunofluorescence
KAP	Knowledge, attitudes, and practices
LD	Lyme disease
NAQ	Nouvelle-Aquitaine
OR	Odds ratio
PCR	Polymerase chain reaction
VETs	Veterinarians
WSAVA	World Small Animal Veterinary Association
