# Supplementary material for: The Challenge of Lyme Borreliosis: Knowledge, Attitudes, and Practices in France
Source: Biology (Basel). 2025 Sep 17;14(9):1286. doi: 10.3390/biology14091286 (PMC12467624; doi:10.3390/biology14091286)
Supplement: Supplementary file 1 [file biology-14-01286-s001.zip › Questionnaire S1 EN.pdf]

The Challenge of Lyme Borreliosis: Knowledge, Attitudes, and Practices in France

Lyme disease (Lyme borreliosis) is caused by bacteria belonging to the Borrelia burgdorferi complex and affects many animals, including humans. It is very widespread in North America and Europe, is constantly increasing, and fits perfectly within the One Health concept (“One health”) (Cosson, 2019).

This study aims to raise public awareness about this zoonosis (1) by investigating the behaviors and knowledge of individuals and professionals.

For this, we invite you to complete a questionnaire mainly intended for pet owners, which should take you about 10 minutes. If you do not own a dog or a cat, you can still answer the questionnaire; it will simply be shorter. Your responses are very important and will help us to understand and perceive the perceptions, habits and practices of pet owners in relation to Lyme borreliosis.

This questionnaire is anonymous and confidential. The results will be used only for the purposes of this study.

We thank you in advance for your cooperation.

(1) The World Health Organization defines a zoonosis as "an infectious disease that has passed from an animal to humans. Zoonotic pathogens can be bacterial, viral or parasitic in origin, or may involve unconventional agents, and can be transmitted to humans through direct contact or through food, water or the environment." Source: https://www.who.int/fr/news-room/fact-sheets/detail/zoonoses

Cosson, J. F. (2019). Ecology of Lyme Disease. Santé Publique, 31, 73-87. https://doi.org/10.3917/spub.190.0073

*Required

I. Validation

1. Do you agree to answer this questionnaire? *

Only provide one answer.

▢ Yes

▢ No (Proceed to section 8 – Thank you for your participation.)

2. Do you agree that the information obtained may be used in this study? *

Only provide one answer.

▢ Yes

▢ No (Proceed to section V – *Thank you for your participation.*)

II. Owner

3. You are: *

Only provide one answer.

▢ A man

▢ A woman

▢ Other

4. Into which age group do you fall? *

Only provide one answer.

▢ Under 18 years

▢ 18 to 29 years

▢ 30 to 49 years

▢ 50 to 65 years

▢ Over 65 years

5. What is your education level? *

Only provide one answer.

▢ Level 3 **vocational secondary education**

▢ Level 4 - high school diploma

▢ Level 5 (**Short-cycle tertiary education)**

▢ Level 6 (Bachelor's Degree)

▢ Level 7 (Master's Degree, Engineering Degree, Specialized Postgraduate Degree)

▢ Level 8 (Doctoral/PhD)

☐ Other: ________

6. What is your socio-professional category? *

Only provide one answer.

☐ Farmers

☐ Craftspeople, shopkeepers and business leaders

☐ Managers and senior intellectual professions

☐ Intermediate professions

☐ Employees

☐ Workers

☐ Retirees

☐ Other people without professional activity

7. Which region do you live in? *

Only provide one answer.

☐ Auvergne-Rhône-Alpes

☐ Bourgogne-Franche-Comté

☐ Bretagne

☐ Centre-Val de Loire

☐ Corse

☐ Grand Est

☐ Hauts-de-France

☐ Île-de-France

☐ Normandie

☐ Nouvelle-Aquitaine

☐ Occitanie

☐ Pays de la Loire

☐ Provence-Alpes-Côte d'Azur

☐ Guadeloupe

☐ Guyane

☐ Martinique

☐ La Réunion

☐ Mayotte

☐ Other______________________

8. In which type of area do you live? *

Only provide one answer.

- ☐ Urban area
- ☐ Peri-urban area
- ☐ Rural area

9. What type of residence do you have? *

Only provide one answer.

- ☐ House
- ☐ Apartment
- ☐ Other: ________

10. Which species do you own? *

Only provide one answer.

- ☐ Dog (Go to question 11)
- ☐ Cat (Go to question 11)
- ☐ Dog and cat (Go to question 11)
- ☐ Other: ________ (Go to question 22)

III. Animal (if dog and/or cat)

11. Does your animal have outdoor access? *

Only provide one answer.

- ☐ Yes
- ☐ No

12. What places does your pet frequent?*

Multiple answers possible.

- ☐ Garden
- ☐ City
- ☐ Forest
- ☐ Lake/riverbank
- ☐ Countryside (meadows and fields)

☐ It does not go outside

☐ Other: ________

13. Do you engage in outdoor activities with your animal (such as hunting, walks in the woods, etc.)?

Only provide one answer.

☐ Yes

☐ No

14. Have you ever seen ticks on your animal? *

Only provide one answer.

☐ Yes

☐ No

15. Have you ever removed ticks from your animal? *

Only provide one answer.

☐ Yes

☐ No (Proceed to question 17)

16. If yes, how did you remove them?

Only provide one answer.

☐ Tick remover (tweezers)

☐ With your hands, without any product

☐ With a product (such as ether, alcohol, etc.)

☐ I don’t remove them

17. Do you consider that you live in a tick-infested region? *

Only provide one answer.

☐ Yes

☐ No

18. What kind of external antiparasitic do you use? *

Multiple answers possible.

☐ Anti-parasite collars

☐ "Spot-on" pipettes

☐ Tablets

☐ None

☐ Other: ________

19. How often do you use external antiparasitics (collar, "spot-on" pipette, tablet, etc.) on your animal?

Only provide one answer.

☐ Every month

☐ Every 3 months

☐ Once a year

☐ Never

☐ Other: ________

20. Do you follow your veterinarian's recommendations regarding vaccination (primary vaccination, booster vaccination) for your animal?

Only provide one answer.

☐ Yes

☐ No

21. Is your pet vaccinated against Lyme disease? *

Only provide one answer.

☐ Yes

☐ No

☐ I don't know

IV. Lyme Disease

22. Do you know how this disease is transmitted? *

Only provide one answer.

☐ Yes

☐ No

23. If so, please indicate the ‘vector’ that, to your knowledge, can transmit this disease.

Vector = organism that transmits the disease

Only provide one answer.

☐ Mosquito

☐ Fly

☐ Rodent

☐ Tick

☐ None

☐ I don’t know

☐ Other: ________

24. Do you think ticks carry the bacteria that causes Lyme disease?

Only provide one answer.

☐ Yes

☐ No

☐ I don’t know

25. Do you think a single tick bite can transmit Lyme disease?

Only provide one answer.

☐ Yes

☐ No

☐ I don’t know

26. Do you think that all ticks carry the bacteria that causes Lyme disease?

Only provide one answer.

☐ Yes

☐ No

☐ I don’t know

27. In your opinion, what are the main symptoms of Lyme disease in dogs?

Only provide one answer.

☐ Asymptomatic (no visible symptoms)

☐ Joint symptoms

☐ Urinary symptoms

☐ Cardiac symptoms

☐ Neurological symptoms

☐ I don't know

☐ Other: ________

28. In your opinion, what is the best way to prevent Lyme disease?

Only provide one answer.

☐ External antiparasitics (collar, "spot-on" pipette, tablet, etc.)

☐ Vaccination

☐ Inspecting the animal after exposure and removing ticks

☐ Combination of antiparasitic and vaccination

☐ Combination of antiparasitic and inspection

☐ I don't know

☐ Other: ________

29. Has your veterinarian ever spoken to you about Lyme disease?

Only provide one answer.

☐ Yes

☐ No

☐ I don't remember

30. If yes, in what context?

Only provide one answer.

☐ Routine (awareness about antiparasitics and/or vaccination)

☐ Presence of ticks on the pet

☐ Suspicion of the disease

☐ Other: ________

31. Have you ever had an animal (dog or cat) affected by Lyme disease? *

Only provide one answer.

☐ Yes

☐ No

☐ I don't remember

32. Before this questionnaire, did you know that Lyme disease is transmitted by ticks?

Only provide one answer.

☐ Yes

☐ No

33. Before this questionnaire, did you know of Lyme disease in animals?

Only provide one answer.

☐ Yes

☐ No

34. Before this questionnaire, did you know that Lyme disease also affects humans?

Only provide one answer.

☐ Yes

☐ No

V. Thank you for your participation.
