# Supplementary material for: The Challenge of Lyme Borreliosis: Knowledge, Attitudes, and Practices in France
Source: Biology (Basel). 2025 Sep 17;14(9):1286. doi: 10.3390/biology14091286 (PMC12467624; doi:10.3390/biology14091286)
Supplement: Supplementary file 1 [file biology-14-01286-s001.zip › Questionnaire S2 EN.pdf]

The Challenge of Lyme Borreliosis: Knowledge, Attitudes and Practices in France

Lyme disease (Lyme borreliosis) is caused by bacteria belonging to the Borrelia burgdorferi complex and affects many animals, including humans. It is very widespread in North America and Europe, is constantly increasing, and fits perfectly within the One Health concept (“One health”) (Cosson, 2019). This study aims to raise public awareness about this zoonosis (1) by investigating the behaviors and knowledge of individuals and professionals.

For this, we invite you to complete a questionnaire mainly intended for veterinarians who have practiced or are currently practicing companion animal medicine, with the aim of understanding which diagnostic and preventive practices are most commonly used and how information regarding this disease and its transmission is communicated to pet owners. This questionnaire takes approximately 10 minutes to complete. Your responses will be of great importance for the achievement of this study’s objective

This questionnaire is anonymous and confidential. The results will be used only for the purposes of this study.

We thank you in advance for your cooperation.

(1) The World Health Organization defines a zoonosis as "an infectious disease that has passed from an animal to humans. Zoonotic pathogens can be bacterial, viral or parasitic in origin, or may involve unconventional agents, and can be transmitted to humans through direct contact or through food, water or the environment." Source: https://www.who.int/fr/news-room/fact-sheets/detail/zoonoses

Cosson, J. F. (2019). Ecology of Lyme Disease. Santé Publique, 31, 73-87. https://doi.org/10.3917/spub.190.0073

*Required

I. Validation

1. Do you agree to answer this questionnaire? *

Only provide one answer.

☐ Yes

☐ No (Proceed to section 8 – Thank you for your participation.)

2. Do you agree that the information obtained may be used in this study? *

Only provide one answer.

☐ Yes

☐ No (Proceed to section V – *Thank you for your participation.*)

II. Veterinarian

3. You are: *

Only provide one answer.

☐ A man

☐ A woman

☐ Other

4. Into which age group do you fall? *

Only provide one answer.

☐ 18 to 29 years

☐ 30 to 49 years

☐ 50 to 65 years

☐ Over 65 years

5. For how long you have been practicing as a veterinarian?*

Only provide one answer.

☐ Less than 1 year

☐ 1–5 years

☐ 5–10 years

☐ 10 years or more

6. In which field do you practice?*

Only provide one answer.

☐ Canine

☐ Mixed (canine/rural; canine/equine)

☐ Other: __________

7. Which region do you practice? *

Only provide one answer.

☐ Auvergne-Rhône-Alpes

☐ Bourgogne-Franche-Comté

☐ Bretagne

☐ Centre-Val de Loire

☐ Corse

☐ Grand Est

☐ Hauts-de-France

☐ Île-de-France

☐ Normandie

☐ Nouvelle-Aquitaine

☐ Occitanie

☐ Pays de la Loire

☐ Provence-Alpes-Côte d'Azur

☐ Guadeloupe

☐ Guyane

☐ Martinique

☐ La Réunion

☐ Mayotte

☐ Other: ______________________

8. Do you consider your region to be rich in ticks?*

Only provide one answer.

☐ Yes

☐ No

9. When you find one or more ticks on an animal, do you perform parasite identification?*

Only provide one answer.

☐ Always

☐ Sometimes

☐ Never

III. Lyme disease (dog and cat)

10. During your career, have you ever suspected one or more cases of Lyme borreliosis in dogs and/or cats?*

Only provide one answer.

☐ Yes

☐ No

11. Do you perform tests to diagnose or rule out Lyme disease in dogs and/or cats?*

Only provide one answer.

☐ Yes → Skip to Question 12

☐ No → Skip to Question 13

IV. Lyme disease testing in dogs and/or cats

12. If yes, on average, how many tests have you performed over the past two years (2021–2022)?*

Only provide one answer.

☐ 0

☐ 1-5

☐ 5-10

☐ More than 10

13. What type of test do you perform to screen for Lyme disease?*

☐ Snap test (e.g., SNAP 4Dx® [IDEXX])

☐ PCR

☐ ELISA

☐ Immunofluorescence

☐ None

☐ Other: __________

14. During your career, have you ever diagnosed one or more cases of Lyme disease in dogs and/or cats?*

Only provide one answer.

☐ Yes

☐ No

15. If yes, how many cases have you diagnosed over the past two years (2021–2022)?*

Only provide one answer.

☐ 1–4 cases

☐ 5–10 cases

☐ More than 10 cases

16. Could you specify the species of the diagnosed cases?

(Please indicate the number of dogs and/or cats separately.)

____

____

____

____

17. When do you suspect Lyme disease?*

Only provide one answer.

☐ Presence and identification of the tick (*Ixodes*)

☐ History of presence in an endemic area

☐ Presence of clinical signs

☐ Case history and clinical signs

☐ Never

☐ Other: __________

18. In your opinion, what are the clinical signs suggestive of Lyme borreliosis?

____

____

____

19. In cases of suspected tick-borne disease, do you systematically perform screening for Lyme disease?*

Only provide one answer.

☐ Yes

☐ No

20. Do you offer Lyme borreliosis screening to your clients?*

Only provide one answer.

☐ Yes

☐ No

21. In which situation do you propose screening for this disease?*

☐ Never

☐ Presence of ticks on the animal

☐ History of presence in an endemic area

☐ Presence of clinical signs

☐ Case history and suggestive clinical signs

☐ Other: __________

22. In the case of intermittent and recurrent arthritis in a dog, is Lyme disease one of your differential diagnoses?*

Only provide one answer.

☐ Yes

☐ No

23. In which situation do you use antibiotic therapy against Lyme disease?*

☐ Presence of ticks on the animal (reported by the owner or during the consultation)

☐ Presence of clinical signs

☐ Animal testing seropositive

☐ Presence of both clinical signs and seropositivity

☐ Other: __________

24. In your opinion, what is the best method of prevention against Lyme disease?*

Only provide one answer.

☐ External antiparasitic treatments (EAP)

☐ Vaccination

☐ Inspection of the animal after exposure and removal of ticks

☐ Combination of EAP and vaccination

☐ Combination of EAP and inspection

☐ Other: __________

25. Do you consider vaccination against Lyme disease to be important?*

Only provide one answer.

☐ Yes, all dogs should be vaccinated

☐ Yes, for animals with high exposure

☐ No

26. If your answer to the previous question is "No", please specify why.

____

____

____

V. Communication with owners

27. Do you talk to your clientele about Lyme disease?*

Only provide one answer.

☐ Yes

☐ No

28. If yes, in what context?*

Only provide one answer.

☐ Routinely (awareness about antiparasitics and/or vaccination)

☐ Presence of ticks on the animal

☐ Suspicion of the disease

☐ Other: __________

29. Do you consider your clientele to be sufficiently informed about Lyme disease (mode of transmission, prevention, etc.)?

- ☐ 0-Not informed at all
- ☐ 1-Slightly informed
- ☐ 2-Moderately informed
- ☐ 3-Well informed
- ☐ 4-Very well informed
- ☐ 5-Completely informed

Thank you for your participation.
