# Supplementary material for: The Challenge of Lyme Borreliosis: Knowledge, Attitudes, and Practices in France
Source: Biology (Basel). 2025 Sep 17;14(9):1286. doi: 10.3390/biology14091286 (PMC12467624; doi:10.3390/biology14091286)
Supplement: Supplementary file 1 [file biology-14-01286-s001.zip › Suplementary File S1.pdf]

Table S1. Sociodemographic variables for the Companion Animal Owner group characterisation

Variable	Classification	Attributes	Codes
**Gender**	Nominal category	Male	1
		Female	2
		Other (specify)	3
**Age**	Simple numerical interval	< 18 years	1
		18 – 29 years	2
		30 – 49 years	3
		50 – 65 years	4
		> 65 years	5
**Education level**	Nominal category	Level 3 (vocational secondary education)	1
		Level 4 (high school diploma)	2
		Level 5 (Short-cycle tertiary education)	3
		Level 6 (Bachelor's Degree)	4
		Level 7 (Master's Degree, Engineering Degree, Specialized Postgraduate Degree)	5
		Level 8 (Doctoral/PhD)	6
		Other (specify)	7
**Profession**	Nominal category	Farmers	1
		Craftsmen, traders, and entrepreneurs	2
		Executives and intellectual professions	3
		Intermediate professions	4
		Employees	5
		Manual workers	6
		Retired	7
		Other without professional activity	8
**Region**	Nominal category	Auvergne-Rhône-Alpes	1

		Bourgogne-Franche-Comté	2
		Brittany	3
		Centre-Val de Loire	4
		Corsica	5
		Grand Est	6
		Hauts-de-France	7
		Île-de-France	8
		Normandy	9
		Nouvelle-Aquitaine	10
		Occitania	11
		Pays de la Loire	12
		Provence-Alpes-Côte d'Azur	13
		Guadeloupe	14
		French Guiana	15
		Martinique	16
		Réunion	17
		Mayotte	18
		Other (specify)	19
**Area**	Nominal category	Urban area	1
		Periurban area	2
		Rural area	3
**Residence**	Nominal category	House	1
		Apartment	2
		Other (specify)	3
**Animals**	Nominal category	Dog	1
		Cat	2
		Dog and cat	3
		Other (specify)	4

Table S2. Descriptive variables for veterinarian group characterization

Variable	Classification	Attributes	Codes
**Gender**	Nominal category	Male	1
		Female	2
		Other (specify)	3
**Age**	Simple numerical interval	< 18 years	1
		18 – 29 years	2
		30 – 49 years	3
		50 – 65 years	4
		> 65 years	5
**Years in practice**	Nominal category	< 1 year	1
		1 - < 5 years	2
		5 - 10 years	3
		> 10 years	4
**Area(s) of clinical activity**	Nominal category	Small animals	1
		Mixed (Small and large animals)	2
		Other	3
**Region**	Nominal category	Auvergne-Rhône-Alpes	1
		Bourgogne-Franche-Comté	2
		Brittany	3
		Centre-Val de Loire	4
		Corsica	5
		Grand Est	6
		Hauts-de-France	7
		Île-de-France	8
		Normandy	9
		Nouvelle-Aquitaine	10
		Occitania	11
		Pays de la Loire	12
		Provence-Alpes-Côte d’Azur	13
		Guadeloupe	14
		French Guiana	15
		Martinique	16

	Réunion	17
	Mayotte	18
	Other (specify)	19

Table S3. Characterization of Companion Animal Owners Based on Pet Outdoor Access and Activity Patterns.

Variable	Classification	Attributes	Codes
**Outdoor access**	Dichotomous category	Yes	1
		No	2
**Visited locations**	Dichotomous category	Garden – Yes	1
		Garden – No	2
		City – Yes	1
		City – No	2
		Forest – Yes	1
		Forest – No	2
		Lakeside/Riverside – Yes	1
		Lakeside/Riverside – No	2
		Countryside – Yes	1
		Countryside – No	2
		No regular outdoor activity – Yes	1
		No regular outdoor activity – No	2
		Other – Yes	1
		Other – No	2
**Outdoor activities**	Dichotomous category	Yes	1
		No	2

Table S4. Characterization of Individuals Based on Tick-Related Practices.

Variable	Classification	Attributes	Codes
**Tick observations on animal**	Dichotomous category	Yes	1
		No	2
**Tick removal**	Dichotomous category	Yes	1
		No	2
**Removal method**	Nominal category	Tick tweezers	1
		Bare hands without product	2
		With a product (e.g. ether, alcohol...)	3
**Tick-abundant region**	Dichotomous category	Yes	1
		No	2

Table S5. Determination of Preventive Measures Used by the Study Group.

Variable	Classification	Attributes	Codes
**Type of external antiparasitics (EAP)**	Dichotomous category	Collar – Yes	1
		Collar – No	2
		Spot-on – Yes	1
		Spot-on – No	2
		Tablet – Yes	1
		Tablet – No	2
		None – Yes	1
		None – No	2
		Other – Yes	1
		Other – No	2
**EAP frequency**	Nominal category	Every month	1
		Every 2–3 months	2
		Once a year	3
		Never	4
		Other (specify)	5
**Veterinary recommendations**	Dichotomous category	Yes	1
		No	2
**LD vaccination**	Nominal category	Yes	1
		No	2
		Don’t know	3

Table S6. Characterization of Veterinarians Regarding Tick-Related Habits

Variable	Classification	Attributes	Codes
**Tick-abundant region**	Dichotomous category	Yes	1
		No	2
**Tick identification**	Nominal category	Always	1
		Sometimes	2
		Never	3

Table S7. Assessment of Public Knowledge Regarding the Epidemiological Role of Ticks in Lyme Disease (LD).

Variable	Classification	Attributes	Codes
**Disease transmission agent**	Dichotomous category	Yes	1
		No	2
**Vector**	Nominal category	Mosquito	1
		Fly	2
		Rodent	3
		Tick	4
		None	5
		Don’t know	6
		Other (specify)	7
**Ticks carry Lyme agent**	Nominal category	Yes	1
		No	2
		Don’t know	3
**Single bite transmits LD**	Nominal category	Yes	1
		No	2
		Don’t know	3
**All ticks carry Lyme agent**	Nominal category	Yes	1
		No	2
		Don’t know	3

Table S8. Assessment of Public Knowledge Regarding Lyme Disease (LD) in Companion Animals.

Variable	Classification	Attributes	Codes
**Symptoms**	Nominal category	Asymptomatic	1
		Articular	2
		Urinary	3
		Cardiac	4
		Neurological	5
		Don't know	6
		Other (specify)	7
**Prevention**	Nominal category	EPA	1
		Vaccine	2
		Physical inspection of animal	3
		EPA + vaccine	4
		EPA + inspection	5
		Don't know	6
		Other (specify)	7

Table S9. Preventive Measures and Attitudes Toward Vaccination in the Study Group.

Variable	Classification	Attributes	Codes
**Prevention**	Nominal category	EAP	1
		Vaccine	2
		Animal inspection	3
		EAP + vaccine	4
		EAP + inspection	5
		Don't know	6
		Other (specify)	7
**Importance of vaccination**	Nominal category	Yes, for all dogs	1
		Yes, for some	2
		No	3
**Asymptomatic dog**	Dichotomous category	Yes	1
		No	2
**Lack of information**	Dichotomous category	Yes	1
		No	2
**Low prevalence**	Dichotomous category	Yes	1
		No	2
**Parasite control is more important**	Dichotomous category	Yes	1
		No	2
**Low efficacy**	Dichotomous category	Yes	1
		No	2

Table S10. Characterization of Veterinarians Regarding Practices Related to Lyme Disease (LD).

Variable		Classification	Attributes		Codes
**LD suspicion**		Dichotomous category	Yes		1
			No		2
**Diagnostic tests**		Dichotomous category	Yes		1
			No		2
**Number of tests?**		Nominal category	0		1
			1 – 5		2
			5 – 10		3
			> 10		4
**Test type**		Dichotomous category	Snap-test	Yes	1
				No	2
			PCR	Yes	1
				No	2
			ELISA	Yes	1
				No	2
			Immunofluorescence	Yes	1
				No	2
**Antibiotic case**	Tick presence	Dichotomous category	Yes		1
			No		2
	Clinical signs	Dichotomous category	Yes		1
			No		2
	Seropositive animal	Dichotomous category	Yes		1
			No		2
	Signs and seropositivity	Dichotomous category	Yes		1
			No		2
	Other	Dichotomous category	Yes		1
			No		2
**Clinical signs**	Neurological	Dichotomous category	Yes		1
			No		2
	Anorexia	Dichotomous category	Yes		1
			No		2
	Erythema	Dichotomous category	Yes		1
			No		2
	Acute/chronic pain	Dichotomous category	Yes		1
			No		2
	Renal	Dichotomous category	Yes		1
			No		2
	Cardiac	Dichotomous category	Yes		1
			No		2
	Myopathy	Dichotomous category	Yes		1
			No		2
	Apathy	Dichotomous category	Yes		1
			No		2

	Mono/polyarthritis	Dichotomous category	Yes	1
			No	2
	Fever	Dichotomous category	Yes	1
			No	2
	Limping	Dichotomous category	Yes	1
			No	2
	Lymphadenopathy	Dichotomous category	Yes	1
			No	2
	Asymptomatic	Dichotomous category	Yes	1
			No	2
	Tremors	Dichotomous category	Yes	1
			No	2
	Hematological changes	Dichotomous category	Yes	1
			No	2
	Other	Dichotomous category	Yes	1
			No	2
	Don't know	Dichotomous category	Yes	1
			No	2
**Intermittent/ recurrent arthritis = differential diagnosis?**		Dichotomous category	Yes	1
			No	2
**Diagnoses performed**		Dichotomous category	Yes	1
			No	2
**Number of diagnoses?**		Nominal category	0	1
			1 – 4	2
			5 – 10	3
			> 10	4
**Species diagnosed**		Nominal category	None	1
			Dog	2
			Dog and cat	3
**LD suspicion based on:**		Nominal category	Tick presence/identification	1
			Travel history in endemic area	2
			Clinical signs	3
			History + signs	4
			Never	5
			Other (specify)	6

Table S11. Evaluation of Communication and Exposure to Information About Lyme Borreliosis, by CAOs.

Variable	Classification	Attributes	Codes
**Communication**	Nominal category	Yes	1
		No	2
		Don't remember	3
**Context**	Nominal category	Routine consultation	1
		Tick presence	2
		Suspected LD	3
		Other (specify)	4
**Animal with LD**	Nominal category	Yes	1
		No	2
		Don't remember	3

Table S12. Evaluation of Veterinarian Communication Regarding Lyme Borreliosis.

Variable	Classification	Attributes	Codes
**Communication**	Dichotomous category	Yes	1
		No	2
**Context**	Nominal category	Routine consultation	1
		Tick presence	2
		Suspected disease	3
		Other	4
**Public information**	Simple numeric	0	0
		1	1
		2	2
		3	3
		4	4
		5	5

Table S13. Assessment of Public Knowledge Regarding Lyme Disease (LD) in general

Variable	Classification	Attributes	Codes
**LD and ticks**	Dichotomous category	Yes	1
		No	2
**LD and animals**	Dichotomous category	Yes	1
		No	2
**LD in humans**	Dichotomous category	Yes	1
		No	2
